# Intranasal racemic ketamine maintenance therapy for patients with treatment-resistant depression: a naturalistic feasibility study

**DOI:** 10.1186/s12888-024-06448-x

**Published:** 2025-01-07

**Authors:** Katelyn Halpape, Raelle Pashovitz, Annabelle Wanson, Monika Hooper, Evyn M. Peters

**Affiliations:** 1 Department of Psychiatry, College of Medicine, University of Saskatchewan, Saskatoon, SK Canada; 2 College of Pharmacy and Nutrition, University of Saskatchewan, Saskatoon, SK Canada

**Keywords:** Ketamine, Major depressive disorder, Mood disorders, Observational study

## Abstract

**Background:**

Ketamine is a promising therapy for treatment-resistant depression due to its rapid onset, although benefits are often transitory, with patients needing maintenance therapy to prevent relapse. Most data supporting ketamine for treatment-resistant depression refers to the intravenous route of administration, leaving alternative routes lacking in data, especially as maintenance regimens. Moreover, the safety of ketamine maintenance therapy is poorly defined. This report aims to describe and evaluate a novel hospital-to-outpatient intranasal racemic ketamine maintenance therapy program.

**Methods:**

This was an observational program evaluation study. Participants were adult inpatients with treatment-resistant depression who had been successfully treated with intranasal racemic ketamine in hospital and were being referred for outpatient maintenance therapy with an intranasal racemic ketamine spray, administered at a specialized community treatment centre. Effectiveness was assessed with the Self-Report Quick Inventory of Depressive Symptomatology, the Quality of Life Scale, and the Clinical Global Impression-Improvement scale.

**Results:**

Five patients were enrolled, completing up to 14 treatment sessions over 192 days. The mean dose administered throughout treatment was 220 mg (100 to 400 mg). All patients had decreased (or stable) depressive symptoms and increased (or stable) quality of life. There were no serious adverse events or discontinuations due to adverse effects. Reported adverse effects included anxiety and nausea. Slight blood pressure increases were seen during treatment, none of which required intervention.

**Conclusions:**

Intranasal racemic ketamine maintenance therapy for treatment-resistant depression appeared to be feasible and well tolerated, although effectiveness conclusions cannot be drawn from this small pilot study. Further investigations regarding the safety and effectiveness of intranasal ketamine maintenance therapy are warranted.

## Background

Major depressive disorder (MDD) is a leading cause of disability globally [1]. Conventional oral antidepressants have limited efficacy, as a significant number of patients fail to achieve remission, as well as a delayed onset, with full benefits taking weeks to be realized [2]. The N-methyl-D-aspartate (NMDA) receptor antagonist ketamine has shown strong antidepressant and anti-suicidal effects with a rapid onset within hours to a few days [3]. However, benefits provided by ketamine are often transitory, necessitating ongoing maintenance therapy to prevent relapse [4]. 

Most data supporting ketamine therapy for treatment resistant depression (TRD) refers to intravenous (IV) administration [4]. This leaves outstanding questions regarding the safety and effectiveness of more convenient and less invasive routes of administration, such as intranasal (IN), especially when administered as long-term maintenance therapy [4]. Although IN esketamine has long-term safety and efficacy data, there are concerns esketamine may be less effective than racemic ketamine [4]. An additional concern with ketamine therapy for TRD is the associated cost. As an uninsured medical service in Canada, most patients must pay the full cost of treatment in a community setting, ranging from 500 to 750 CAD per treatment [5, 6]. This is a substantial cost that is prohibitively expensive for many patients with TRD. The bioavailability of IN racemic ketamine varies significantly and has been reported to range between 8 and 45% with a time to maximum concentration (T_max_) of 10–20 min [7]. 

Here we report results from an observational program evaluation study surrounding a novel hospital-to-outpatient IN racemic ketamine maintenance therapy program. The purpose of the treatment program was to provide up to 6 months of ketamine maintenance therapy to patients with TRD who were successfully treated with IN ketamine in hospital and were being referred for ongoing maintenance treatment in the community. The purpose of this program evaluation study was to gauge safety and feasibility in terms of preventing depression relapse and hospital readmission, with a focus on patient satisfaction and quality of life.

## Methods

### Participants

Participants were adult inpatients with TRD (unipolar or bipolar) who had been successfully treated with IN ketamine according to a previously published treatment protocol developed for use in a teaching hospital in Saskatoon, Canada [8]. Diagnoses were confirmed prior to starting inpatient ketamine therapy with a structured interview, a clinician-rated depression scale, and patient-reported screening questionnaires for comorbid conditions [8]. Treatment resistance was defined as an inadequate response to two or more antidepressant trials of adequate dose and duration, in the current episode. Patients were eligible to enter this study if their response to inpatient ketamine therapy resulted in a planned discharge from hospital and they were being referred by their inpatient psychiatrist for ongoing maintenance therapy in the community. This study was approved by the University of Saskatchewan Biomedical Research Ethics Board (#3222) and was conducted in accordance with the Tri-Council Policy Statement (TCPS 2). All participants provided written informed consent to participate.

### Procedure

Prospective participants were recruited 1–2 working days before discharge and followed for up to 6 months of maintenance therapy provided in a flexible manner as per existing clinic protocols. Participation in this study had no impact on treatment beyond data collection and the cost of treatment. All costs were covered by a grant funding novel mental health community treatment programs. Consent to treatment with ketamine was obtained separately by the psychiatrist at the community treatment centre, as per usual clinic practices.

For this study, participants completed the Self-Report Quick Inventory of Depressive Symptomatology (QIDS-SR-16) and the Quality of Life Scale (QOLS) prior to each treatment session [9, 10]. After each treatment session, the clinic psychiatrist and registered nurse recorded the dose, baseline and maximum post-treatment blood pressures, adverse effects, medications administered during treatment, pertinent information related to the disposition of the patient (e.g., withdrawing from treatment) or changes to the treatment protocol (e.g., dose or frequency), and whether the patient had any emergency room visits or hospitalizations due to depression since the last treatment session. After six months (or upon early discontinuation) the clinic psychiatrist completed an exit interview with the patient that included the Clinical Global Impression-Improvement (CGI-I) scale and Yes/No questions about patient satisfaction [11]. 

### Ketamine therapy

The outpatient ketamine maintenance therapy utilized racemic ketamine compounded into an intranasal spray of 100 mg/ml (50 mg/spray). The spray was delivered via a mucosal atomization device (MAD). Doses were administered as 0.5 ml (50 mg) sprays into alternating nostrils every 5 min. Patients often self-administered their doses using the MAD, which was overseen by a registered nurse to ensure proper administration technique. If the patient was unable to administer the drug correctly, the registered nurse would administer the dose for the patient. The patients were positioned at 45-degree angles for administration. Vitals, including blood pressure and heart rate, were taken prior to administration, then repeated every 5 min before the next round of spray was delivered. Following administration of the full dose, vitals were repeated after 40 min, during peak onset, then finally, another 50 min later. If vital signs and level of consciousness (LOC) were normal after the 90-minute observation window, the treatment session was concluded. If there were vital sign or LOC abnormalities, patients were monitored until resolution.

## Results

### Patient characteristics

Data was collected from five adult female patients (age 21 to 36 years) with unipolar major depression (*n* = 4) or bipolar depression (*n* = 1). Comorbid diagnoses included anxiety disorders (*n* = 4), PTSD (*n* = 3), OCD (*n* = 1), ADHD (*n* = 2), and chronic pain (*n* = 1). All patients were medically stable with no acute/untreated medical conditions. Concurrent medications were antidepressants (*n* = 5), atypical antipsychotics (*n* = 4), zopiclone (*n* = 3), stimulants (*n* = 2), benzodiazepines (*n* = 2), and lamotrigine (*n* = 1). The average length of the current episode and the duration of hospital stay was 5.5 months and 10.8 days, respectively. The length of time from hospital discharge to first maintenance IN ketamine treatment (study entry) ranged from 8 to 22 days (*M* = 14.2).

### Treatment characteristics

Four of the five patients completed a full course of maintenance therapy (12 to 14 treatment sessions each, over 149 to 192 days). One patient discontinued treatment at 61 days due to relocation. The average interval between treatments was 13.4 days overall; on a per-patient basis, the average interval ranged from 8.71 to 17.5 days. All patients started at approximate bi-weekly dosing; two eventually transitioned to weekly, and overall three patients were treated within a week of the previous dose at least one time throughout the study. Dosage information is presented in Table 1. All patients received dose increases throughout the study period to maintain therapeutic benefits, at the discretion of the treating psychiatrist.

**Table 1:** Descriptive statistics for study the variables

**Dosage**	
Initial dose, *M* mg (range)	110 (100–150)
Final dose, *M* mg (range)	330 (200–400)
Average dose across all treatments, *M* mg (*SE*)	220 (17.0)
**Effectiveness Questionnaires**	
QIDS-SR-16 initial score, *M* (range)	21.4 (19–24)
QIDS-SR-16 score decrease by final session, *M* (range)	7.8 (3–14)
QOLS initial score, *M* (range)	48.6 (37–55)
QOLS score increase by final session, *M* (range)	10.4 (0–36)
CGI-I score at the final session, *M* (range)	1.8 (1–3)
**Blood Pressures**	
SBP change across all treatments, *M* mmHg (*SE*)	8.98 (1.96)
DBP change across all treatments, *M* mmHg (*SE*)	8.27 (1.75)
**Patient Satisfaction Questions**	
Satisfied with treatment overall, % answered “Yes”	80%
Benefits outweighed negatives, % answered “Yes”	80%
Would continue with treatment, % answered “Yes”	100%
Bothered by side effects during treatment, % answered “No”	100%
Any bothersome side effects between treatments, % answered “No”	100%

Note. Standard errors are robust, adjusting for clustering at the subject level. *M* = mean; QIDS-SR-16 = Self-Report Quick Inventory of Depressive Symptomatology; QOLS = Quality of Life Scale; CGI-I = Clinical Global Impression Scale – Improvement; SBP = systolic blood pressure; DBP = diastolic blood pressure

### Treatment outcomes

From baseline to the final session, all patients experienced a reduction in QIDS-SR-16 scores and an increase in QOLS scores (Table 1). Regarding readmission to hospital, one patient was hospitalized briefly after having missed two sessions for logistical reasons. Another was hospitalized but remained stable post-discharge for two more months once ketamine treatment frequency increased from bi-weekly to weekly. At the final exit interview, no CGI-I scores indicated worsening depression throughout the maintenance period (i.e., no score > 3) and most patients reported satisfaction with treatment (Table 1).

### Adverse effects and tolerability

There were no serious adverse events or discontinuations due to adverse effects. There was one incident of anxiety, which did not require intervention. There were seven occurrences of nausea in two patients who were usually treated with antiemetics (e.g., ondansetron, dimenhydrinate). One patient missed a treatment session after developing urinary tract symptoms that were investigated and found to be unrelated to ketamine; treatment resumed in one week and continued for three months without the symptoms returning. Reports at the final session suggested satisfactory tolerability (Table 1). Blood pressure increases were seen (Table 1), none of which required intervention.

## Discussion

As far as we are aware, this is the first prospective study documenting the feasibility of IN ketamine maintenance therapy for patients with TRD. While effectiveness conclusions cannot be drawn from this small pilot study, all patients experienced decreased (or stable) depressive symptoms and increased (or stable) quality of life during the study period, with minimal adverse effects and no patient tolerability concerns. This is also the first prospective study documenting the safety and tolerability of IN ketamine for TRD at doses above 100 mg. This is noteworthy, since a previous trial of 100 mg IN had to be discontinued early due to adverse effects after only three patients had been treated with ketamine [12]. Of note, these patients were much older (age 52, 60, and 64) compared to our patients who had already demonstrated adequate tolerability to IN ketamine (max dose 75 mg) in hospital [8]. 

While all patients had improved or unchanged depressive symptoms during maintenance therapy, significant dose increases were made throughout the treatment period (and in some cases, treatment frequency was also increased). This could indicate tachyphylaxis. Although doses above 0.5 mg/kg are generally not thought to be more effective for acute induction treatment, some patients undergoing maintenance therapy in naturalistic settings seem to require higher doses [13, 14]. Alternatively, since QIDS-SR-16 scores continued to decrease during maintenance treatment, it is possible that higher doses could have provided additional benefits if administered earlier. Furthermore, QIDS-SR-16 scores were quite high by the first treatment, suggesting some degree of symptom relapse following hospital discharge and prior to initiating maintenance. Patients were enrolled in this study if they responded to IN ketamine as an inpatient; however, depression scores at hospital discharge were not captured. Nonetheless, even if some patients had been under-treated or relapsed prior to starting maintenance therapy, it is not clear that ongoing treatment beyond six months would have continued to be a viable option had more dose increases been needed. This is perhaps one reason to be less sanguine about the results.

A limitation of this pilot study was the small sample size. We initially planned to recruit 10 to 12 patients, but changes in provincial mandates regulating ketamine therapy intensified the monitoring and staffing requirements for IN ketamine therapy (i.e., anaesthesiologists required for all ketamine administration regardless of route). Consequently, this greatly increased the cost of treatment such that our funding was no longer sufficient for the planned sample size. Part of the rationale for establishing this program was that IN ketamine could be delivered in community treatment centres at less than half the cost per treatment compared to IV ketamine. However, costs were subsequently equated by these regulatory changes. From a clinical standpoint, our pilot results were reasonably promising, but without a cost advantage, it is hard to justify the ongoing clinical use of IN over IV ketamine considering the stronger evidence base supporting the latter. Another limitation is that no data was captured for other treatment changes during the study period. Dissociation was not routinely measured, although it was not reported as an adverse effect by any of the patients and did not necessitate any treatment modifications. Despite these limitations, we believe that further investigations regarding the safety and efficacy of IN ketamine therapy are warranted—it is already being used in clinical practice, and there are still likely cost advantages in other jurisdictions that could significantly benefit TRD patients for whom IV ketamine therapy is unaffordable [13]. 

## Conclusions

Intranasal racemic ketamine maintenance therapy for TRD appeared to be a feasible treatment option for patients successfully treated with IN ketamine in hospital. Doses ranging from 100 to 400 mg were well tolerated without any major adverse effects. Effectiveness conclusions cannot be drawn from this small pilot study, thus, further investigations regarding the safety and effectiveness of IN ketamine maintenance therapy are warranted.

## Data Availability

The data that support these findings are not publicly available due to ethical restrictions (i.e., containing information that could compromise the privacy of research participants).

## Abbreviations

ADHD: Attention-deficit hyperactivity disorder
CGI-I: Clinical Global Impression-Improvement
IN: Intranasal
IV: Intravenous
LOC: Level of consciousness
MAD: Mucosal atomization device
MDD: Major depressive disorder
NMDA: N-methyl-D-aspartate receptor
OCD: Obsessive compulsive disorder
PTSD: Post-traumatic stress disorder
QIDS-SR-16: Self-Report Quick Inventory of Depressive Symptomatology
QOLS: Quality of Life Scale
TRD: Treatment-resistant depression
